# The comparison of the commonly used surrogates for baseline renal function in acute kidney injury diagnosis and staging

**DOI:** 10.1186/s12882-016-0220-z

**Published:** 2016-01-09

**Authors:** Charat Thongprayoon, Wisit Cheungpasitporn, Andrew M. Harrison, Wonngarm Kittanamongkolchai, Patompong Ungprasert, Narat Srivali, Abbasali Akhoundi, Kianoush B. Kashani

**Affiliations:** 1 Division of Nephrology and Hypertension, Mayo Clinic, 200 First Street SW, Rochester, MN 55905 USA; 2 Medical Scientist Training Program, Mayo Clinic, Rochester, MN USA; 3 Division of Rheumatology, Mayo Clinic, Rochester, MN USA; 4 Division of Pulmonary and Critical Care Medicine, Mayo Clinic, Rochester, MN USA

**Keywords:** Acute kidney injury, Back-calculation, Creatinine, Estimated creatinine, Mortality

## Abstract

**Background:**

Baseline serum creatinine (SCr) level is frequently not measured in clinical practice. The aim of this study was to investigate the effect of various methods of baseline SCr determination measurement on accuracy of acute kidney injury (AKI) diagnosis in critically ill patients.

**Methods:**

This was a retrospective cohort study. All adult intensive care unit (ICU) patients admitted at a tertiary referral hospital from January 1, 2011 through December 31, 2011, with at least one measured SCr value during ICU stay, were included in this study. The baseline SCr was considered either an admission SCr (SCr_ADM_) or an estimated SCr, using MDRD formula, based on an assumed glomerular filtration rate (GFR) of 75 ml/min/1.73 m^2^ (SCr_GFR-75_). Determination of AKI was based on the KDIGO SCr criterion. Propensity score to predict the likelihood of missing SCr was used to generate a simulated cohort of 3566 patients with baseline outpatient SCr, who had similar characteristics with patients whose outpatient SCr was not available.

**Results:**

Of 7772 patients, 3504 (45.1 %) did not have baseline outpatient SCr. Among patients without baseline outpatient SCr, AKI was detected in 571 (16.3 %) using the SCr_ADM_ and 997 (28.4 %) using SCr_GFR-75_ (*p* < .001). Compared with non-AKI patients, patients who met AKI only by SCr_ADM_, but not SCr_GFR-75_, were significantly associated with 60-day mortality (OR 2.90; 95 % CI 1.66–4.87), whereas patients who met AKI only by SCr_GFR-75_, but not SCr_ADM_, had a non-significant increase in 60-day mortality risk (OR 1.33; 95 % CI 0.94–1.88). In a simulated cohort of patients with baseline outpatient SCr, SCr_GFR-75_ yielded a higher sensitivity (77.2 vs. 50.5 %) and lower specificity (87.8 vs. 94.8 %) for the AKI diagnosis in comparison with SCr_ADM_.

**Conclusions:**

When baseline outpatient SCr was not available, using SCr_GFR-75_ as surrogate for baseline SCr was found to be more sensitive but less specific for AKI diagnosis compared with using SCr_ADM_. This resulted in higher incidence of AKI with larger likelihood of false-positive cases.

**Electronic supplementary material:**

The online version of this article (doi:10.1186/s12882-016-0220-z) contains supplementary material, which is available to authorized users.

## Background

Acute kidney injury (AKI) is commonly associated with high morbidity and mortality in critically ill patients [1–4]. Even a modest degree of AKI is associated with mortality, morbidity, and increased healthcare cost [5–10]. The Kidney Disease: Improving Global Outcomes (KDIGO) criteria was developed and validated to standardize the diagnosis and severity of AKI based on absolute or relative increases from baseline serum creatinine (SCr) levels, as well as progressive oliguria [11]. Thus, determination of baseline SCr is important to diagnose and classify AKI [12, 13].

Despite development of the KDIGO criteria, classification of AKI remains challenging, as baseline outpatient SCr measurement—a marker of kidney function prior to the critical illness—is often unavailable [14]. In this case, the Acute Dialysis Quality Initiative (ADQI) has recommended backward calculation of baseline SCr using the Modification of Diet in Renal Disease (MDRD) formula, this formula assumes an estimated glomerular filtration rate (eGFR) value of 75 ml/min/1.73 m^2^ (SCr_GFR-75_) for all patients with missing data [15]. However, backward calculation can lead to misclassification of AKI, particularly in the early stages of this syndrome [16, 17].

The European Renal Best Practice (ERBP) recently proposed using the first documented SCr value on hospital admission (SCr_ADM_)—rather than SCr_GFR-75_—as the baseline SCr when baseline outpatient SCr measurements are missing [18]. However, the use of SCr_ADM_ as the baseline SCr can be inaccurate in patients with community-acquired AKI, as the SCr may have already increased prior to hospitalization [19, 20]. Additionally, the predictive performance of these two methods on mortality has not been well studied [18].

The primary objective of this study was to compare the incidence and staging of AKI according to SCr criteria using SCr_GFR-75_ versus SCr_ADM_. The secondary objective of this study was to determine the accuracy of AKI diagnosis in critically ill patients using SCr estimation, based on assumed GFR and SCr_ADM_, compared to the reference standard of SCr measurement.

## Methods

### Study Population

This is a single-center retrospective study conducted at a tertiary referral hospital. We studied all adult patients (age ≥18 years) admitted to the ICU at Mayo Clinic in Rochester, MN, from January 1, 2011 through December 31, 2011. We included patients who had at least one SCr measured during the ICU admission. Patients with a history of stage 5 chronic kidney disease (CKD) or end-stage renal disease (ESRD), patients who received any dialysis modalities within 14 days prior to the ICU admission, and those who did not provide research authorization were excluded from the study. Stage 5 CKD and ESRD were identified based on ICD-9 code assignment (Additional file 1: Table S1) or baseline outpatient SCr-calculated eGFR of <15 ml/min/1.73 m^2^. For patients with multiple ICU admissions, only the first ICU admission during the study period was included in the analysis. This study was approved by the Mayo Clinic institutional review board.

**Fig. 1:**
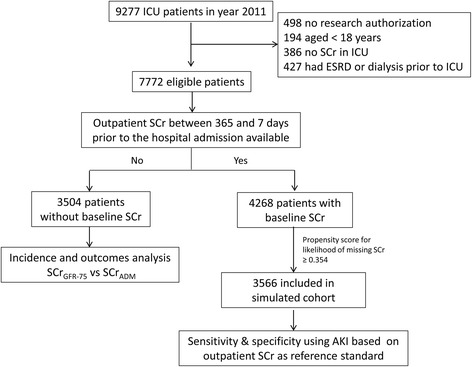
Study inclusion and exclusion flow diagram

We divided eligible patients into two groups, based on the availability of outpatient SCr between 365 and 7 days prior to hospital admission (Fig. 1). One group included patients without baseline outpatient SCr (*n* = 3504). This is the cohort of patients for whom the use of surrogates for baseline SCr was actually applied. This cohort was used to compare the incidence and outcomes of AKI using SCr_GFR-75_ versus SCr_ADM_ in order to represent the real-world finding. The second group included patients with baseline outpatient SCr (*n* = 4268).

### Simulated cohort

AKI diagnosis based on outpatient SCr baseline can be used as a reference standard to assess the sensitivity and specificity of AKI diagnosis using various surrogate estimates. However, due to significant differences in clinical characteristics between patients with and without outpatient SCr (Table 1), the finding in the cohort of patients with outpatient SCr might not be generalizable to patients without outpatient SCr. Therefore, we developed a propensity score model in which the outcome was whether baseline SCr was missing (*n* = 3504) or not (*n* = 4268) to predict the likelihood of missing baseline SCr. The propensity model included age, race, diabetes mellitus, hypertension, coronary artery disease, stroke, peripheral vascular disease, congestive heart failure, and APACHE III at ICU admission as covariates. We applied this model to the group of patients with baseline outpatient SCr and then selected patients with a propensity score of ≥0.354 (post-hoc cut-off) to generate a study cohort of patients with similar characteristics to patients whose baseline SCr was not available, while preserving known baseline SCr (*n* = 3566). We used this simulated cohort to analyze the sensitivity and specificity of AKI diagnosis based on different surrogates. AKI diagnosis based on outpatient SCr was used as the reference standard.

**Table 1:** Clinical characteristics and outcomes of eligible critically ill patients admitted to the ICU during the study period

Characteristics	Outpatient SCr not available	Outpatient SCr available	p*	Simulated Cohort^a^	p*
N	3,504	4,268		3566	
Age, year	62 ± 18	65 ± 16	<0.001	63 ± 16	0.05
Male sex	2013 (57)	2508 (59)	0.24	2033 (57)	0.71
White	3246 (93)	4039 (95)	<0.001	3347 (94)	0.10
Baseline creatinine, mg/dL					
- Admission SCr	1.1 ± 0.9	1.1 ± 0.8	0.55	1.1 ± 0.8	0.10
- Single imputation of GFR 75 ml/min/1.73 m^2^	1.0 ± 0.1	1.0 ± 0.1	0.20	1.0 ± 0.1	0.06
- Baseline outpatient SCr	-	1.1 ± 0.4	-	1.1 ± 0.4	-
Comorbidities					
- Diabetes Mellitus	723 (21)	1114 (26)	<0.001	730 (20)	0.87
- Hypertension	1836 (52)	2653 (62)	<0.001	2004 (56)	0.04
- Coronary artery disease	809 (23)	1376 (32)	<0.001	793 (22)	0.39
- Stroke	272 (8)	472 (11)	<0.001	240 (7)	0.12
- Peripheral vascular disease	120 (3)	258 (6)	<0.001	60 (2)	0.10
- Congestive heart failure	311 (9)	588(14)	<0.001	252 (7)	0.07
Medical ICU type	1733 (49)	2036 (48)	0.12	1689 (47)	0.08
APACHE III	44 ± 22	50 ± 20	<0.001	46 ± 18	0.06
Renal replacement therapy use in ICU	89 (3)	109 (3)	0.97	78 (2)	0.33
ICU mortality	102 (3)	136 (3)	0.48	94 (3)	0.48
60-day mortality	298 (9)	468 (11)	<0.001	338 (9)	0.15

*Abbreviations*: *APACHE* Acute Physiology and Chronic Health Evaluation, *GFR* glomerular filtration rate, *ICU* intensive care unit, *IQR* interquartile range *SCr* serum creatinine, *SOFA* sequential organ failure assessment

*Compared with patients without baseline outpatient SCr

^a^Simulated cohort was a subset of patients with available baseline outpatient SCr with propensity score to predict the likelihood of missing baseline SCr of ≥ 0.354

Continuous data are presented as mean ± SD, categorical data are presented as N (%), if not indicated

### Data collection

Clinical characteristics, demographic information, and laboratory data were collected using manual and automated retrieval from the institutional electronic medical records. We utilized data from the Mayo Clinic Life Science System (MCLSS) and the Multidisciplinary Epidemiology and Translational Research in Intensive Care (METRIC) database. The MCLSS database contained demographic characteristics, clinical data, hospital admission information, diagnosis codes, procedure codes, laboratory test results, and flowsheet data of both in- and outpatients at our institution [21]. The METRIC database contained ICU admission information, pertinent vital signs, fluid input/output, and medication administration record data of all patients admitted in ICU at our institution [22]. Data variables collected included age, sex, race, known comorbidities at hospital admission, ICU type, severity of illness at ICU admission, ICU length of stay, and ICU discharge status. SCr measurements were collected for each eligible patient up to one year prior to ICU admission. The baseline outpatient SCr was defined as the most recent outpatient SCr measured between 365 and 7 days prior to the hospital admission. The eGFR was derived using the MDRD equation, with CKD being defined as a calculated eGFR of <60 ml/min/1.73 m^2^. We identified comorbidities from clinical notes in electronic medical record using a validated electronic note search strategy [21]. The severity of illness at ICU admission was evaluated using the Acute Physiology and Chronic Health Evaluation (APACHE) III score [23].

### AKI diagnosis and classification

We identified and staged AKI based solely on the SCr criterion of the KDIGO definition [11]. AKI was defined as an increase in SCr in the ICU of ≥0.3 mg/dL or relative change of ≥50 % from the baseline. The baseline SCr was calculated using two different methods; 1) the first SCr available during hospital admission (SCr_ADM_), and 2) an estimated SCr using the MDRD formula, based on an assumed GFR of 75 ml/min/1.73 m^2^ (SCr_GFR-75_, as recommended by the ADQI working group). We used the following equation for backward calculation of SCr_GFR-75_:

$$ \mathrm{S}\mathrm{C}{\mathrm{r}}_{\mathrm{GFR}\hbox{-} 75} = {\left(75/\left[186\ *\ \left(\mathrm{ag}{\mathrm{e}}^{\hbox{-} 0.203}\right)\ *\ \left(0.742\ \mathrm{f}\mathrm{o}\mathrm{r}\ \mathrm{women}\right)\ *\ \left(1.21\ \mathrm{f}\mathrm{o}\mathrm{r}\ \mathrm{black}\right)\right]\right)}^{\hbox{-} 0.887} $$

### Clinical outcomes

The primary outcome was all-cause mortality at 60 days following ICU admission. We reviewed patient vital statistics by reviewing the patient registration and electronic medical records. In patients whose vital status at 60 days after ICU admission was unknown, the Social Security Death Index was used [24].

### Statistical analysis

Continuous variables were reported as mean with standard deviation (SD) or median with interquartile range (IQR), as appropriate. All categorical variables were reported as counts with percentages. The difference in the AKI diagnosis using SCr_ADM_ andSCr_GFR-75_ was assessed using McNemar’s test. The agreement of AKI diagnosis and staging, based on SCr_ADM_ and SCr_GFR-75_, was assessed using Cohen’s weighted kappa coefficient with linear weight between AKI stages. According to the results of AKI diagnosis, based on SCr_ADM_ and SCr_GFR-75_, we classified patients into 4 groups: (1) patients who had AKI regardless of baseline SCr calculation method, (2) patients who had AKI based only on SCr_ADM_, (3) patients who had AKI only based on SCr_GFR-75_, and (4) patients who did not have AKI, regardless of baseline SCr determination methodology. We adjusted the odds ratio (OR) for age, ICU type, and APACHE III scores to assess 60-day mortality for the first three groups, using the fourth group as the reference group. The association between AKI stages and 60-day mortality was assessed using a logistic regression analysis. The predictive performance of the SCr criterion, using SCr_ADM_ and SCr_GFR-75_, for 60-day mortality was assessed by C-statistics, after which we compared their performances using Delong’s test. We calculated net reclassification improvement to evaluate how using SCr_GFR-75_ as baseline renal function for AKI diagnosis changed risk classification for 60-day mortality.

Sensitivities and specificities were compared using McNemar’s test. To determine the optimal GFR used for SCr estimation, we estimated based on an assumed GFR with an increment of 5 ml/min/1.73 m^2^, ranging from 30 to 120 ml/min/1.73 m^2^. The sensitivity and specificity of AKI diagnosis, according to the estimated SCr of each assumed GFR, was calculated using AKI diagnosis according to the baseline outpatient SCr as a reference standard. The Youden index, which yielded the highest sum of sensitivity and specificity, was used to identify the optimal GFR used for SCr estimation. The subgroup analyses, based on sex, age, and the presence of abnormal renal function at presentation, were performed to investigate the optimal GFR used in each subgroup. A two-sided *p* value of less than 0.05 was considered statistically significant. All analyses were performed using JMP statistical software (version 10.0, SAS, Cary, NC).

## Results

During the study period, 9277 critically ill patients were admitted to the ICU. Of these, 1,505 were excluded: 498 did not provide authorization to use their data for research, 194 aged < 18 years, and 386 had no measured SCr values in ICU, 427 had ESRD, or received dialysis within 14 days prior to ICU admission. Thus, 7772 patients were included in this study. The baseline outpatient SCr was not available for 3504 of these patients (45.1 %). The clinical characteristics of these patients upon ICU admission and their outcomes are summarized in Table 1. Patients who had available baseline outpatient SCr were older, Caucasian, had more known comorbidities, and had higher APACHE and SOFA scores at ICU admission. Patients in the simulated cohort had similar characteristics to patients without baseline outpatient SCr.

### AKI diagnosis and staging using the admission and estimated SCr

Among patients without baseline outpatient SCr, using SCr_ADM_, AKI occurred in 571 patients (16.3 %), with 12.1 % in stage 1, 2.3 % in stage 2, and 1.9 % in stage 3. UsingSCr_GFR-75_, AKI occurred in 997 patients (28.4 %), with 15.6 % in stage 1, 7.4 % in stage 2 and 5.5 % in stage 3. SCr_GFR-75_ classified more patients into AKI than SCr_ADM_ (*p* < .001) (Table 2).

**Table 2:** AKI diagnoses and staging using admission SCr and GFR-estimated SCr for patients without baseline outpatient SCr (*n* = 3504)

AKI stage (SCr_GFR-75_)	AKI stage (SCr_ADM_)				Total N (%)
	0	1	2	3	
0	2361 (67.4)	133 (3.8)	13 (0.4)	0 (0)	2507 (71.6)
1	356 (10.2)	160 (4.6)	28 (0.8)	2 (0.1)	546 (15.6)
2	134 (3.8)	85 (2.4)	31 (0.9)	10 (0.3)	260 (7.4)
3	82 (2.3)	45 (1.3)	9 (0.3)	55 (1.6)	191 (5.5)
Total, N (%)	2933 (83.7)	423 (12.1)	81 (2.3)	67 (1.9)	3504 (100)

Kappa = 0.42 (95 % CI 0.39-0.46) and percentage agreement = 79.5 % for AKI diagnosis,

Kappa = 0.39 (95 % CI 0.36-0.42) and percentage agreement = 74.4%for AKI staging

Abbreviation: AKI, acute kidney injury; SCr_ADM_, the admission serum creatinine; SCr_GFR-75_, an estimated serum creatinine based on an assumed GFR of 75 ml/min/1.73 m^2^

The percentage agreement for AKI diagnosis using SCr_ADM_ and SCr_GFR-75_ for baseline SCr estimation was 79.5 % with a kappa of 0.42 (95 % CI, 0.39–0.46). SCr_ADM_ and SCr_GFR-75_ as baseline SCr agreed in 425 AKI cases and 2361 non-AKI cases. Using SCr_ADM_ and SCr_GFR-75_ resulted in a discrepancy in AKI diagnoses of 718 cases (20.5 %). 146 patients met the AKI diagnosis by only SCr_ADM_ and 572 met the AKI diagnosis using only SCr_GFR-75_. The percentage agreement for AKI staging, using both SCr_ADM_ and SCr_GFR-75_, was 74.4 % with a kappa of 0.39 (95 % CI, 0.36–0.42). Ninety six percent of AKI based only by SCr_GFR-75_ but not SCr_ADM_ occurred within 24 hours of ICU admission.

### Risk for 60-day mortality

**Fig. 2:**
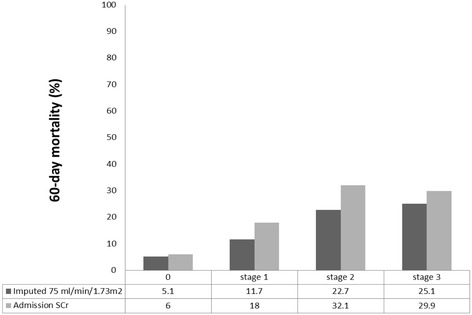
60-day mortality risk stratified by AKI stage in patients without baseline outpatient SCr

**Fig. 3:**
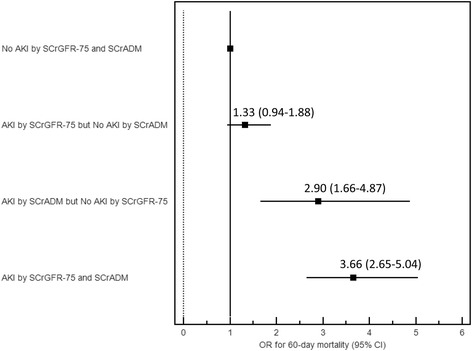
Odds ratio for 60-day mortality in patients with or without acute kidney injury using admission SCr and GFR-estimated SCr as baseline

Of the total cohort, 8.5 % (*N* = 298) died within 60 days of ICU admission. The 60-day mortality rates after ICU admission for AKI stages by using SCr_ADM_ and SCr_GFR-75_ are shown in Fig. 2. Compared with patients without AKI, patients who met AKI regardless of baseline SCr methodology, and patients who met AKI only by SCr_ADM_, but not SCr_GFR-75_, were significantly associated with increased 60-day mortality (OR = 3.66 [95 % CI, 2.65–5.04] and OR = 2.90 [95 % CI, 1.66–4.87]). However, patients who met AKI only by SCr_GFR-75_, but not SCr_ADM_, had a non-significant increase in 60-day mortality risk (OR 1.33; 95 % CI 0.94–1.88) (Table 3 and Fig. 3). Calculating the performance for prediction of 60-day mortality, the C-statistic for AKI stages using SCr_ADM_ and SCr_GFR-75_ as baseline SCr were 0.64 and 0.68 respectively (*p* = .001). Using SCr_GFR-75_ for AKI diagnosis improved risk classification for 60-day mortality with net reclassification improvement of 4.7 %.

**Table 3:** 60-day mortality risk based on the AKI diagnosis using admission and GFR-estimated SCr in patients without baseline outpatient SCr (*n* = 3504)

N 60-day mortality, n (%) ^a^OR (95 % CI)	No AKI-SCr_ADM_	AKI-SCr_ADM_
No AKI- SCr_GFR-75_	2361	146
	4.5 %	13.7 %
	1 (ref)	2.90 (1.66-4.87)
AKI- SCr_GFR-75_	572	425
	12.1 %	24.0 %
	1.33 (0.94-1.88)	3.66 (2.65-5.04)

*Abbreviation*: *AKI* acute kidney injury, *CI* confidence interval, *SCr*_*ADM*_ the admission serum creatinine, *SCr*_*GFR-75*_ an estimated serum creatinine based on an assumed GFR of 75 ml/min/1.73 m^2^

^a^adjusted for age, APACHE III score and ICU type

### Sensitivity and specificity for AKI diagnosis

In the simulated cohort of patients with available baseline outpatient SCr, the mean baseline SCr (SD) was 1.1 ± 0.4 mg/dL. Of these patients, 20.7 % (*n* = 738) experienced AKI using known baseline outpatient SCr, 14.6 % (*n* = 520) using SCr_ADM_, and 25.7 % (*n* = 915) using SCr_GFR-75_. Application of SCr_GFR-75_ as baseline SCr yielded a sensitivity of 77.2 % and specificity of 87.8 % for the AKI diagnosis. Using SCr_ADM_ as baseline SCr yielded a sensitivity of 50.5 % and specificity of 94.8 %. Overall, SCr_GFR-75_ was more sensitive (*p* < .001)_,_ whereas SCr_ADM_ was found to be more specific (*p* < .001). The application of either SCr_GFR-75_ or SCr_ADM_, compared to AKI diagnosis and staging based on baseline outpatient SCr, resulted in bi-directional misclassification of AKI (Additional file 1: Table S2).

### Optimal GFR used for SCr estimation

The sensitivity and specificity of AKI diagnosis, when using different assumed GFR for SCr estimation, were shown in Table 4. The most optimal GFR with the highest sum of sensitivity and specificity was 85 ml/min/1.73 m^2^, yielding a sensitivity of 85.2 % and a specificity of 83.0 %. The sensitivity and specificity in different subgroups based on sex, age, and the presence of abnormal renal function at presentation are summarized in Additional file 1: Table S3 with the most optimal GFR for SCr estimation indicated. The application of SCr_ADM_ for AKI diagnosis in patients with abnormal renal function at presentation resulted in significantly lower sensitivity (41.7 vs. 65 %), but similar specificity (92.7 vs. 95.3 %), for AKI diagnosis.

**Table 4:** The corresponding sensitivities and specificities for AKI diagnosis using an estimated SCr, based on various assumed GFR

SCr_GFR_ (ml/min/1.73 m^2^)	Sensitivity	Specificity
30	26.8	99.5
35	34.0	99.3
40	39.8	98.8
45	47.0	97.8
50	53.0	96.8
55	56.4	95.6
60	63.7	93.9
65	68.8	91.9
70	73.0	90.2
75	77.2	87.8
80	81.7	85.0
85	85.2^a^	83.0^a^
90	87.7	78.5
95	90.5	76.2
100	91.7	73.4
105	94.0	68.7
110	95.7	66.0
115	96.6	63.3
120	97.0	57.0
SCr_ADM_	50.5	94.8

*Abbreviation*: *SCr*_*ADM*_ the admission serum creatinine, *SCr*_*GFR*_ GFR-estimated serum creatinine

^a^surrogate for baseline SCr yielding highest sum of sensitivity and specificity

## Discussion

We conducted a retrospective cohort study to assess the effect of using different baseline SCr methods, SCr_GFR-75_ and SCr_ADM_, on the incidence of AKI as well as prognostication performance of the KDIGO definition. We demonstrated that using SCr_GFR-75_ as a baseline SCr for AKI diagnosis was associated with the larger number of classified AKI cases. However, using SCr_GFR-75_ resulted in the misclassification of non-AKI cases into AKI, as AKI cases only by SCr_GFR-75_, but not SCr_ADM_, had a no significant increase in 60-day mortality risk. This finding is consistent with SCr_GFR-75_ as a more sensitive and SCr_ADM_ as a more specific surrogate for AKI diagnosis when compared with outpatient SCr as the reference standard.

Ideally, outpatient SCr values, which are reflective of patient pre-morbid kidney function, should be used as the baseline SCr for AKI diagnosis [25]. However, the lack of baseline outpatient SCr level is a common problem encountered in clinical practice [25, 26]. Following the ADQI statement, the use of SCr_GFR-75_ as a baseline SCr has been shown to lead to misclassification of AKI in ICU patients [16, 17] and following cardiac surgery [27]. The European Renal Best Practice (ERBP) has recommended the use of SCr_ADM_, rather than SCr_GFR-75_, when baseline outpatient SCr measurements are not available [18]. In our study, we demonstrated that using either SCr_GFR-75_ or SCr_ADM_ led to misclassification of AKI diagnosis and staging. Use of SCr_GFR-75_ has been shown to inflate AKI incidence, whereas use of SCr_ADM_ underestimates AKI incidence [14, 16, 17]. The inflation of AKI diagnosis bySCr_GFR-75_as surrogates for baseline kidney function was due to inaccurate baseline SCr estimation in patients with chronic kidney disease [14, 28]. In contrast, the underestimation of AKI diagnosis when using SCr_ADM_ was due to the under-diagnosis of community-acquired AKI [14]. We found most of AKI cases missed by SCr_ADM_ occurred within the first 24 hours of ICU admission.

The analysis in a simulated cohort of patients with baseline outpatient SCr showed the higher sensitivity and lower specificity of AKI diagnosis bySCr_GFR-75_ in comparison with SCr_ADM._ The decision to use SCr_GFR-75_ or SCr_ADM_ for AKI diagnosis and classification depends on the purpose of the AKI definition. In clinical practice, AKI prevention and prompt treatment might improve patient outcomes. Thus, for risk stratification purposes in clinical practice, we encourage the use of SCr_GFR-75_ for AKI diagnosis, as it can conceivably identify more AKI cases. Conversely, for research studies that enroll patients with AKI for invasive tests or treatments, using SCr_ADM_ may be more suited, as it would be more likely to enroll patients who are going to benefit from the intervention.

The use of estimated SCr by back-calculation with the MDRD formula can allow investigators or clinicians to be more flexible regarding the sensitivity and specificity of the AKI definition. As shown in Table 4 and Additional file 1: Table S3, when using different assumed GFR for SCr estimation, it provides different levels of sensitivity and specificity that we may individualize in each patient in different encounters. Since GFR decreases with age, the use of SCr_GFR-75_ might result in over-AKI classification in the elderly [29], therefore use of a different assumed GFR for SCr estimation could be considered in different age groups. For example, using SCr_GFR-70_ in elderly but SCr_GFR-100_ in younger adult would yield the highest sum of sensitivity and specificity. In addition, abnormal renal function at presentation may be due to pre-existing chronic kidney disease or due to acute elevation of SCr from community-acquired acute kidney injury. Therefore, estimated SCr based on lower assumed GFR might be more suitable for patients without baseline outpatient SCr, who present initially with an abnormal SCr. In contrast, using SCr_ADM_ in patients with abnormal renal function based on initial SCr value in hospital would result in lower sensitivity in the diagnosis of AKI.

This study has several limitations. (1) This is a single-center retrospective study. (2) We did not include the urine output criterion for AKI diagnosis since an indwelling urinary catheter was not used to obtain accurate hourly urine output data for all critically ill patients. (3) Our study compared only two common surrogates for baseline SCr and did not include other advanced methods to estimate the baseline SCr. For example, Siew and colleagues recently demonstrated that a multiple imputation method can improve accuracy in estimating missing baseline SCr and reduce misclassification of AKI [30]. However, the use of this technique in clinical practice is limited and still requires further validation. A multi-center prospective study is ultimately required to address some of these limitations.

## Conclusion

When baseline outpatient SCr was not available, using SCr_GFR-75_ as a surrogate for baseline SCr was found to be more sensitive but less specific for AKI diagnosis compared to SCr_ADM_. This resulted in higher incidence of AKI with larger likelihood of false-positive cases.

## Additional file

Additional file 1: Table S1. ICD-9 codes used for detection of end-stage renal disease. **Table S2:** AKI diagnoses and staging using the admission SCr and GFR-estimated SCr compared to AKI diagnoses and staging based on baseline outpatient SCr. **Table S3:** Sensitivities and specificities for AKI diagnosis using an estimated SCr, based on various assumed GFR in subgroups of patients (DOCX 28 kb)

## Abbreviations

AKI: Acute kidney injury
CI: Confidence interval
GFR: Glomerular filtration rate
ICU: Intensive care unit
IQR: Interquartile range
SCr: Serum creatinine
SCr_ADM_: Admission serum creatinine
SCr_GFR-75_: Estimated serum creatinine, using MDRD formula, based on an assumed glomerular filtration rate of 75 ml/min/1.73 m^2^
