# Supplementary material for: The comparison of the commonly used surrogates for baseline renal function in acute kidney injury diagnosis and staging
Source: BMC Nephrol. 2016 Jan 9;17:6. doi: 10.1186/s12882-016-0220-z (PMC4707008; doi:10.1186/s12882-016-0220-z)
Supplement: Additional file 1: Table S1. — ICD-9 codes used for detection of end-stage renal disease. Table S2: AKI diagnoses and staging using the admission SCr and GFR-estimated SCr compared to AKI diagnoses and staging based on baseline outpatient SCr. Table S3: Sensitivities and specificities for AKI diagnosis using an estimated SCr, based on various assumed GFR in subgroups of patients (DOCX 28 kb) [file 12882_2016_220_MOESM1_ESM.docx]

Table S1: ICD-9 codes used for detection of end-stage renal disease

| ICD-9 | Condition |
| --- | --- |
| 585.5 | Chronic Kidney Disease, stage V |
| 585.6 | End Stage Renal Disease |
| 996.73 | Other complications due to renal dialysis device implant and graft |
| 996.68 | Infection and inflammatory reaction due to peritoneal dialysis catheter |
| 996.56 | Mechanical complication due to peritoneal dialysis catheter |
| 792.5 | Cloudy (hemodialysis or peritoneal dialysis) effluent |
| 458.21 | Hypotension of dialysis |

Table S2: AKI diagnoses and staging using the admission SCr and GFR-estimated SCr compared to AKI diagnoses and staging based on baseline outpatient SCr

1. Admission SCr

| AKI stage  (SCr_ADM_) | AKI stage (baseline outpatient SCr) | | | | Total  N (%) |
| --- | --- | --- | --- | --- | --- |
|  | 0 | 1 | 2 | 3 |  |
| 0 | 2681 (75.2) | 285 (8.0) | 47 (1.3) | 33 (0.9) | 3046 (85.4) |
| 1 | 143 (4.0) | 194 (5.4) | 50 (1.4) | 14 (0.4) | 401 (11.3) |
| 2 | 4 (0.1) | 17 (0.5) | 29 (0.8) | 12 (0.3) | 62 (1.7) |
| 3 | 0 (0) | 0 (0) | 5 (0.1) | 52 (1.5) | 57 (1.6) |
| Total, N (%) | 2828 (79.3) | 496 (13.9) | 131 (3.7) | 111 (3.1) | 3566 (100) |

Sensitivity 50.5%; Specificity 94.8%

1. Imputed SCr, assuming GFR of 75 m/min/1.73 m^2^

| AKI stage  (SCr_GFR-75_) | AKI stage (baseline outpatient SCr) | | | | Total  N (%) |
| --- | --- | --- | --- | --- | --- |
|  | 0 | 1 | 2 | 3 |  |
| 0 | 2483 (69.6) | 161 (4.5) | 7 (0.2) | 0 (0) | 2651 (74.3) |
| 1 | 306 (8.6) | 217 (6.1) | 44 (1.2) | 4 (0.1) | 571 (16.0) |
| 2 | 36 (1.0) | 90 (2.5) | 54 (1.5) | 21 (0.6) | 201 (5.6) |
| 3 | 3 (0.1) | 28 (0.8) | 26 (0.7) | 86 (2.4) | 143 (4.0) |
| Total, N (%) | 2828 (79.3) | 496 (13.9) | 131 (3.7) | 111 (3.1) | 3566 (100) |

Sensitivity 77.2%; Specificity 87.8%

Abbreviation: AKI, acute kidney injury; SCr_ADM_, the admission serum creatinine; SCr_GFR-75_, an estimated serum creatinine based on an assumed GFR of 75 ml/min/1.73m^2^

Table S3: Sensitivities and specificities for AKI diagnosis using an estimated SCr, based on various assumed GFR in subgroups of patients

| SCr_GFR_  (ml/min/1.73 m^2^) | Sex | | | | | Age | | | | ^#^GFR < 60 ml/min/1.73 m^2^ | | | |
| --- | --- | --- | --- | --- | --- | --- | --- | --- | --- | --- | --- | --- | --- |
|  | Male | | | Female | | <70 years | | ≥70 years | | No | | Yes | |
|  | Sensitivity | Specificity | | Sensitivity | Specificity | Sensitivity | Specificity | Sensitivity | Specificity | Sensitivity | Specificity | Sensitivity | Specificity |
| 30 | 25.9 | | 99.4 | 28.2 | 99.7 | 24.5 | 99.5 | 31.3 | 99.5 | 9.3 | 100.0 | 37.6 | 97.2 |
| 35 | 33.3 | | 99.2 | 35.0 | 99.4 | 31.3 | 99.3 | 39.3 | 99.3 | 12.5 | 100.0 | 47.2 | 96.1 |
| 40 | 38.7 | | 98.9 | 41.5 | 98.7 | 37.2 | 98.8 | 44.8 | 98.7 | 15.7 | 100.0 | 54.6 | 93.3 |
| 45 | 46.0 | | 97.9 | 48.6 | 97.7 | 43.6 | 98.2 | 53.6 | 97.1 | 21.1 | 100.0 | 62.9 | 87.8 |
| 50 | 50.5 | | 97.0 | 56.8 | 96.5 | 49.4 | 97.4 | 59.9 | 95.6 | 26.4 | 100.0 | 69.2* | 82.1* |
| 55 | 53.8 | | 95.9 | 60.2 | 95.2 | 52.5 | 96.6 | 63.9 | 93.9 | 29.6 | 99.9 | 72.7 | 75.5 |
| 60 | 60.8 | | 94.2 | 68.0 | 93.5 | 58.4 | 95.6 | 73.8 | 90.9 | 34.6 | 99.8 | 81.4 | 66.5 |
| 65 | 67.1 | | 91.6 | 71.4 | 92.3 | 63.2 | 94.7 | 79.8 | 87.0 | 39.3 | 99.6 | 86.9 | 56.6 |
| 70 | 71.4 | | 89.7 | 75.5 | 90.8 | 67.3 | 93.9 | 84.1* | 83.6* | 44.3 | 99.3 | 90.6 | 48.3 |
| 75 | 76.4 | | 87.3 | 78.6 | 88.5 | 72.8 | 92.0 | 85.7 | 80.5 | 50.4 | 98.5 | 93.7 | 39.1 |
| 80 | 81.3 | | 83.5 | 82.3 | 87.1 | 78.6 | 89.6 | 87.7 | 77.0 | 58.6 | 97.2 | 95.9 | 29.4 |
| 85 | 85.4* | | 81.2* | 85.1* | 85.2* | 82.7 | 88.1 | 90.1 | 73.9 | 65.7 | 95.7 | 97.2 | 24.9 |
| 90 | 87.2 | | 76.3 | 88.4 | 81.4 | 85.0 | 84.9 | 92.9 | 67.4 | 71.4 | 92.3 | 97.6 | 15.2 |
| 95 | 90.1 | | 74.0 | 91.2 | 79.1 | 89.3 | 81.3 | 92.9 | 67.3 | 78.6 | 89.8 | 97.8 | 14.0 |
| 100 | 92.1 | | 69.9 | 91.2 | 77.8 | 90.3* | 80.9* | 94.4 | 60.3 | 81.1 | 87.0 | 98.3 | 11.1 |
| 105 | 94.8 | | 65.4 | 92.9 | 72.9 | 92.4 | 77.1 | 97.2 | 54.1 | 86.1 | 81.9 | 98.9 | 8.1 |
| 110 | 95.9 | | 62.7 | 95.2 | 70.2 | 94.9 | 72.9 | 97.2 | 55.0 | 90.0* | 78.7* | 99.1 | 7.9 |
| 115 | 96.9 | | 59.8 | 96.3 | 67.8 | 95.9 | 71.4 | 98.0 | 49.2 | 91.8 | 75.6 | 99.6 | 7.3 |
| 120 | 97.3 | | 53.6 | 96.6 | 61.3 | 96.5 | 67.4 | 98.0 | 38.9 | 92.5 | 68.6 | 99.8 | 4.1 |
| SCr_ADM_ | 51.8 | | 94.5 | 48.6 | 95.2 | 50.8 | 95.9 | 50.0 | 92.9 | 65.0 | 95.3 | 41.7 | 92.7 |

^#^GFR was calculated based on admission SCr using MDRD formula

*surrogate for baseline SCr yielding highest sum of sensitivity and specificity

Abbreviation:SCr_ADM_, the admission serum creatinine; SCr_GFR_, an GFR-estimated serum creatinine
